# Ring-Enhancing Lesions in the Brain: A Diagnostic Dilemma

**Published:** 2014

**Authors:** Guruprasada SHETTY, Kadke Shreedhara AVABRATHA, Boodyar Sanjeev RAI

**Affiliations:** 1 Department of Pediatrics, Father Muller Medical College, MangaloreKarnataka, India

**Keywords:** Magnetic resonance spectroscopy, Neurocysticercosis, Tuberculoma

## Abstract

The most common radiological abnormality seen in young Indian patients with epilepsy is single small enhancing (ring/disc) computed tomographic (CT) lesions. The two most common differential diagnosis of this lesion in clinical practice include neurocysticercosis (NCC) and tuberculomas. They have similar clinical and neuroimaging features. Few researchers believe that in poor and developing countries (where both tuberculosis and NCC are common) that it is difficult to differentiate between tuberculomas and a single cysticercal granulomas.

We report a case of a 6-year-old female patient who presented with complex partial seizures. The patient’s neuroimaging showed a single ring-enhancing lesion in the brain that was not differentiated between NCC and Tuberculoma.

Finally, Magnetic resonance spectroscopy (MRS) was suggestive of tuberculoma that was successfully treated with antituberculosis therapy.

This report highlights diagnostic difficulties with conventional investigations in single ring enhancing lesions in the brain and role of MRS in a diagnosis.MRS is helpful in differentiating these two conditions.

## Introduction

A ring-enhancing lesion in brain imaging is a common feature on the Indian subcontinent. The two most common etiologies of inflammatory granulomas encountered in pediatric clinical practice include neurocysticercosis (NCC) and tuberculomas (1).The size, shape, wall thickness of ring-enhancing lesions, the extent of surrounding edema, and, more importantly, clinical history andage of the patient should be taken into consideration to help distinguish the condition (2).

Hence, we report a 6-year-old female patient who presented with a ring-enhancing lesion in brain and a conventional investigation failed to properly diagnose.

However, MRS helped us to arrive at the correct diagnosis. This report highlights the diagnostic difficulties with conventional investigations in single ring enhancing lesion in the brain and the role of MRS in the diagnosis.

## Case history

A 6-year-old female patient with normal development presented with sudden onset multiple episodes of right sided simple partial seizures with secondary generalization.

There was no history of fever, headache, or vomiting. There was no history of contact with tuberculosisor epilepsy in the family. The patient was born to middle class, nonconsanguineous parents.

On examination, the patient weighed 16kg and was104 cm in height. BCG scars were present. There were no neurocutaneous markers or subcutaneous nodules. 

Systemic and neurological examination was normal. Blood counts including ESR and chest X ray were normal and the Mantoux test was negative. An electroencephalogram (EEG) revealed left parietal-posterior and temporo-occipital delta wave dysfunction. Magnetic resonance imaging(MRI)of the brain showed well defined lobulated racemose ring enhancing altered signal intensity lesion(10–14mm in size) noted in the left occipital lobe with perilesional edema, which was suggestive of infective granuloma either tuberculoma or NCC (Fig.1A and 1B). There was no evidence of papill edema/intraocular cysticercosis on ophthalmological examination. A confirmation of the diagnosis used an MRS of the lesion that identified increases in the choline/ creatine ratio and decreases in N-acetylaspartate (NAA) to indicate a diagnosis that favors tuberculoma. 

Hence, the diagnosis of tuberculoma was considered. The patient was subsequently started on a 4drug antitubercular drug therapy (2HRZE+10HR) with oral steroids for 8 weeks with additional conventional antiepileptic drug added. The patient responded to the antituberculosis therapy and no further episodes of convulsion have been noted. A repeat MRI scan done at 6 months revealed total disappearance of the lesion (Fig.2A and 2B).

## Discussion

The most common neuroimaging finding in children with partial epilepsy from Indiais single enhancing lesions (SEL) with perifocal density suggestive of edema(3, 4).Wadia et al reported that 26.1% of Indian patients with focal seizures had enhancing ring or disc lesions visible in CT scans (5).The etiological spectrum of SEL in Indiaseems to be different from that described in the Western literature with infections like NCC and tuberculomas likely to be significant causes of ring-enhancing lesions. With the introduction of HIV/AIDS, toxoplasmosis, and fungal infections such as cryptococcosis or histoplasmosis are increasingly associated with ring enhancement as well. Other causes for ring-enhancement are primary brain tumors, metastases, brain abscesses, granulomas, resolving hematomas, and infarcts (2).

In developing countries, most of these SEL are caused by NCC or tuberculosis with differential diagnoses extremely difficult to ascertain. This is because the clinical and imaging features are similar. These diseases are common in endemic areas and may coexist in the same patient (3). Attempts have been made to differentiate between NCC and tuberculoma based on clinical assessment and imaging features. Common characteristics of cysticerci are round in shape, 20 mm (or smaller) in size, and with ring enhancement or visible scolex. In addition, cerebral edema is severe enough to produce midline shifts or focal neurological deficits are not visible. Tuberculomas, by contrast, are usually irregularly shaped, solid, and greater than 20 mm in size. 

**Fig 1A:**
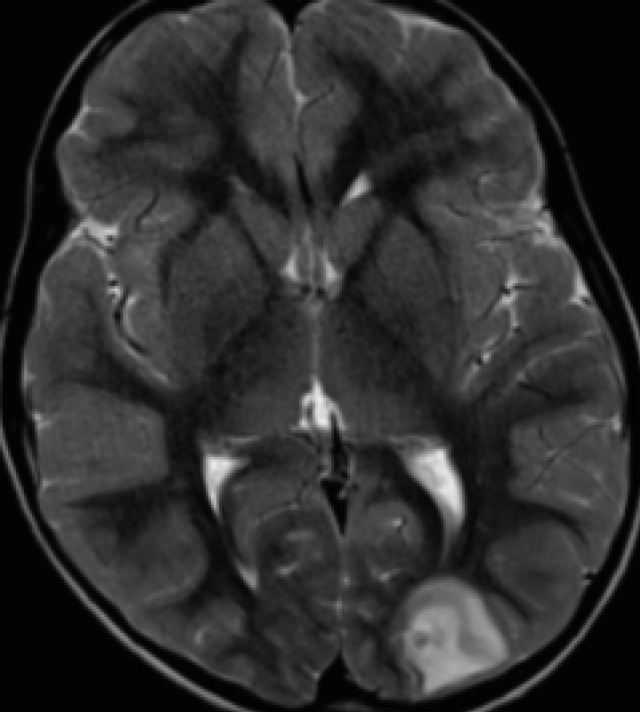
Axial T2 weighted image showing welldefined lobulated racemose ring lesion (10–14mm in size) in the left occipital lobe

**Fig 1B:**
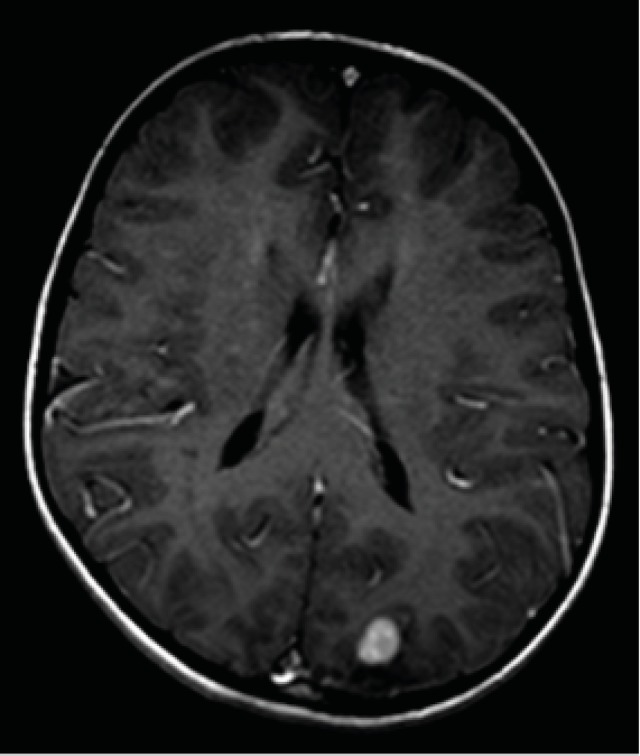
The same lesion showed enhancement after contrast with perilesional edema

**Fig 2A:**
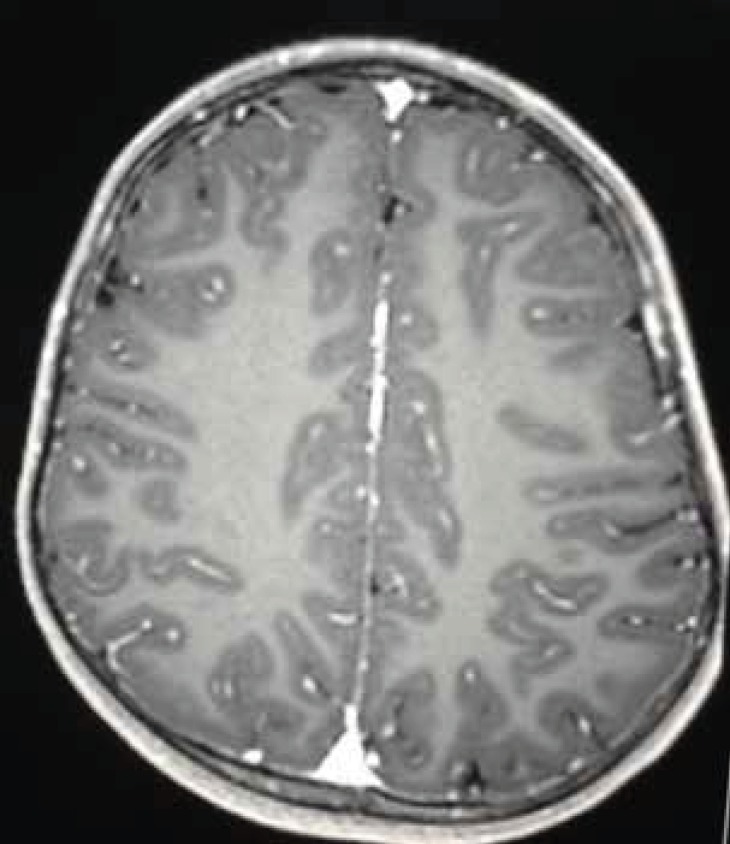
Axial T2 - A repeated MRI after 6months showing complete resolution

**Fig 2B:**
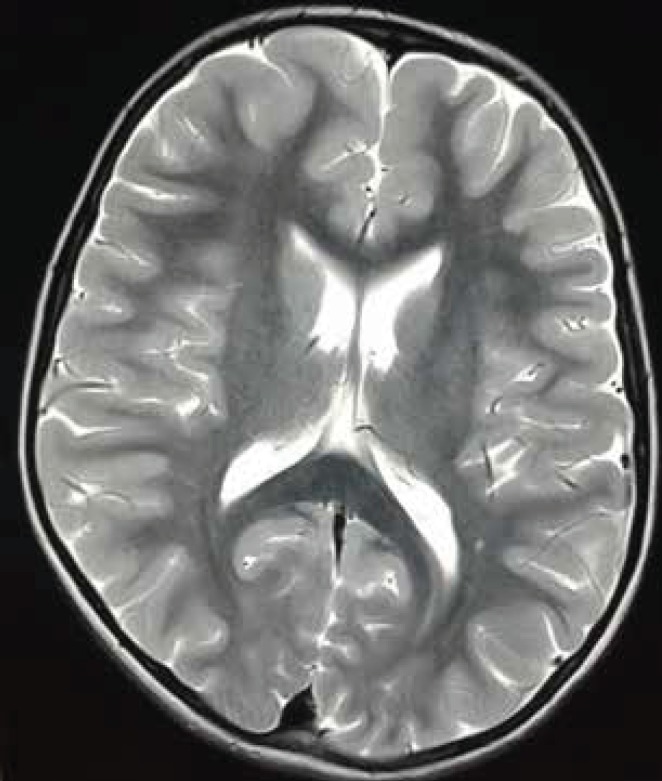
(post contrast) –A repeat MRI after 6months

They are often associated with severe perifocal edema and focal neurological deficits (1, 3, 6). The MRI is frequently performed with the objective of differentiating between cysticercal granuloma and tuberculoma.In fact, the MRI features of both the seconditions are also similar and usually not useful for differentiation.

MRS complements the MRI as a non-invasive means for the characterization of the tissue. While the MRI uses signals from hydrogen protons to form anatomic images, the proton MRS uses this information to determine the concentration of brain metabolites such as N-acetyl aspartate (NAA), choline (Cho), creatine (Cr), and lactate in the tissue examined. MRS offers a promising investigation technique to differentiate tuberculoma from other infective granulomas (7,9).

An MRS of brain tuberculomas commonly detects peaks of lipids attributable to large lipid fractions in tuberculosis bacillus. It will also have increased choline levels and decreased N acetyl aspartate and creatine levels. The choline/ creatine ratio was greater than 1 in alltuberculomas but not in cysticerci (1,8,9).The Proton-MRS allows for non-invasive identification of tuberculoma with high specificity and may allow early instalment of targeted antimicrobial treatment (9,10). 

In our case study, an MRI was inconclusive but the MRS indicated a more favorable diagnosis of tuberculoma.

Hence, the patient was started on ATT. The patient responded well to the treatment and fully recovered.

**In conclusion, **the distinction between NCC and tuberculoma is important because parenchymal cysticercosis is a benignand self-limiting condition, whereas tuberculoma is anactive infection requiring prolonged therapy that involvespotentially toxic drugs. 

## Author’s contribution

Guruprasada Shetty: Collecting data

Kadke Hreedhara Avabratha: Review literature

Boodyar Sanjeev Rai: Preparing manuscript
